# Parental mediation of smart device use and its impact on language development in early childhood

**DOI:** 10.1038/s41598-026-38833-9

**Published:** 2026-02-10

**Authors:** Zaid Alkouri, Faleh Jadan Aldhafeeri

**Affiliations:** 1 Department of Educational Sciences, Princess Alia University College, Al-Balqa Applied University, Shmeisani, Amman, Jordan; 2 Department of Psychology, College of Humanities and Social Sciences, Northern Border University, Arar, Saudi Arabia

**Keywords:** Parental mediation, Smart devices, Interaction, Language development, Communication, Cultural and media studies, Cultural and media studies, Education, Psychology, Psychology

## Abstract

This study investigated the mediation strategies that parents use regarding the use of smart devices and how those strategies can subsequently affect the enhancement of language in children in Amman. The study applied a stratified sampling technique in its recruitment of 82 families from Amman. The theoretical underpinning of the study was laid by Vygotsky’s Zone of Proximal Development Theory, proposing that active mediation on the part of the parents would create the use of smart devices for interaction and thus help in the acquisition of a language. Instead, the discovery revealed that parents were using passive mediation, that is, rules of when and how screens should be used, rather than active control and guidance. The screen time duration rule, which in many cases involves no direct interaction, can be a difficult to the development of optimum language skills. The research found another very remarkable point: there was no agreement by parents on whether content held educational benefits for using the smart device to foster early reading and language skills, even under their supervision. This doubt is also attached to the value of the parents’ involvement in the promotion of linguistic learning and understanding the context in which language operates. The study based on these results strongly recommends that future efforts in Jordan concentrate on developing and implementing target educational programs for parents. This program should highlight the critical role of active parental mediation and emphasize more on practices such as co-viewing and interaction during smart device use to stimulate early language development effectively.

**Supplementary Information:**

The online version contains supplementary material available at 10.1038/s41598-026-38833-9.

## Introduction

Smart devices play a vital role in daily life. An electronic gadget linked to the Internet can perform tasks such as remote control, and tasks much beyond its basic function, if able-also voice commands. Typically having microprocessors, and networking capability, it can collect data, share information, and operate autonomously. Intelligent smart devices are daily somethings with progressive computing, including artificial intelligence and machine learning, and networking as part of the Internet of Things^1^. Electronic gadgets that connect to other devices or networks can be termed as smart devices. They can connect with other devices or networks and can function as well as communicate with other devices or networks. They can function and decide upon some limited options by themselves based on information and algorithms^2^. Smart devices can interact with humans through touch screens, voices, or sensors, and they can collect environmental or user data. Most of the time, they can collect and analyze such data for the betterment of their functions. Such devices are mostly controllable and monitor able using internet applications^3^. There are some examples of smart devices such as smartphones, smart watches, smart TVs, smart locks, smart home security systems, smart refrigerators, and smart cars^4^.

Smart devices, particularly smartphones, are heavily used by children as a medium of communication and entertainment. Smart devices are increasingly important today since they bring a convenience, efficiency, and security to daily life. They automate the provision of some service or routine task and subsequently provide an individual with an option to remotely control his or her home environment, like lighting, heating, or appliances, with minimal effort, usually voice-activated or through smartphone apps^5^.

Parents should establish a healthy balance, both for themselves and their children, in using smartphones, tablets, and the like, and involve their children in open discussions to help them understand the reasons for setting such rules. Nowadays, children are used to being busy with smart devices. They are spread in every house, and the children engage themselves in certain activities. Unguided device usage may cause risks to language development by reducing opportunities for interactive, face-to-face communication. Parental mediation surely plays an important role in shaping how children can use smart devices, mitigating negative effects, and fostering language-rich interactions. Despite growing concern, limited practical research has investigated how parental mediation particularly affects the relations between smart devises use and language development in early childhood.

The settings rules for screen time, app use, and proper online behavior, which essentially lead children to develop proper digital habits, such as being accustomed to some screen-free periods for, say, meals and an hour before sleeping. With this, parents can now talk frankly and without being judgmental about the experiences of their children with digital engagements^6,7^. They must be informed about the risks of such things as cyberbullying and misuse, and other harmful activities that children can involve themselves in. Being a good role model by also using the devices correctly adds to the habit formation, as in most cases, children tend to follow what their parents do. The effective parental mediation should not be viewed only from the perspective of control but rather as an active and collaborative process of setting limits. Such a strategy as co-using devices for educational purposes and allowing the children to participate in setting rules enhances the relationships between the parents and children, which could protect against problematic smartphone use^8^. Monitoring by parents must also be balanced with the autonomy of the child at this level of adolescence. Encouraging children to use the internet safely and responsibly, and with proper positive behavior, creates a learning environment rather than an authoritarian regime, allowing for balanced and healthy device use. This form of mediation, which is participatory and respectful, is very effective in promoting balanced and healthy device use^9^.

### Problem statement

The problem statement emanates from the upsurge in the use of smart tools by parents within Amman, which has elicited concerns over the nature of parental mediation of the technology and its effects on the early childhood development of language. There are few studies that have been conducted into determining the specific nature of the use of and mediation strategies on children’s acquisition of language during this important period in their development.

### Research objective and question

The main research objective of this paper is to investigate the effects of varying modes of parental mediation over the use of smart devices by children on the development of language skills among pre-adolescents in Amman. This aim attempts to explain how the strategies of parental mediation contribute to offsetting the enhancement or deteriorating effects of the exposure of smart devices on the skills of children of that age. The main research question of this study is: How do parents mediate smart device use about its outcomes on language improvement in early childhood in the Amman area?

### Research significance

This study is significant as it covers the main role of parental mediation over the use of smart devices and its direct effect on the development of early childhood language, a principal development area affected by the increasing digital exposure in Jordan. Such dynamics help parents, educators, and policymakers learn in what way the use of smart devices can be made the best use of to support, not to stand in the way of the little ones in acquiring language in a fast-digitalizing world.

### Brief outline of the article structure

The first section of this research will introduce why parental mediation for managing young children’s use of smart devices may be important and how it could affect the developing language of young children in Amman. Then, a summary of the literature will follow that examines the various forms of parental mediation, the exposure of young children to smart devices, and the effects of such exposure to smart devices on the language development of young children. The methodology of this study covers research design, selection of participants, and data collection, comprising parental mediation practices and assessments of language development. The study closes with results presentations that also discuss the implications regarding the enhancement of the skills of language development and recommendations for the parents and policy-makers. All this has to be done in the right environment of the Amman region.

## Literature review

### Theoretical framework

The theoretical underpinning of the research concerns how parents guide young children in using smart technology and the implications for developing children’s language skills. This is based on Parental Mediation Theory on regulating and directing older children as they use smart technology. These were expected to agree with outcomes on developmental outcomes, especially language skills. The technological advancement is very helpful in bringing information to all strata of society. This development has to be taken up wisely and put to use in an efficient manner so that all can benefit^10^. It is the parents, particularly those who are completely in charge of childhood, because of the failure or success of a child. A child is considered to be like a blank slate on which the value of becoming a masterpiece is determined, and therefore, the success point within the learning process of the child mostly depends on how it is handled by the parents^11^. Parental behaviors, such as joint media engagement and interactional turn-taking, where the first one is active and involves communication between the parents and the child on media content, are positively related to children’s language development, including vocabulary, grammar, and pragmatic skills^12^. The use of a smartphone by parents in child routines can lead to technology, the mere presence of which reduces the quality of parent–child interactions by decreasing responsiveness, following the gaze, and joint attention, which are all preconditions for language acquisition^13^. Therefore, the framework underlines that quality and context in parental mediation have to have a say in the good order, not the mere availability of smart devices that play a fundamental role in early language development by either facilitating or impeding real-life linguistic interaction between the child and the adult.

Smart devices have become a core part of the daily lives of children, effectively a member of the nuclear family. Parental mediation of media is crucial in helping children to properly comprehend media messages and have a healthy relationship with smart devices^14^. The parental mediation plays a vital role in guiding the children’s interactions with smart devices, more particularly smartphones and tablets. Conscious and effective management of their smart devices by the children could be ensured by parents, who, in turn, apply proper mediation strategies^12^. A good theoretical base is also needed to determine how effective parental mediation is mainly for the form of the children’s media interactions and the level of guidance that is available from the parents. Parental mediation is defined as the behaviors and strategies of parents that maximize the positive effects of smart devices on children and minimize the negatives^8^. Najiha et al. discussed the importance of the parent to stimulate the language development in their child through their broad thinking skills and ability to communicate well, as shown by studies on children’s language development^15^. The children who have access to smart devices in early childhood have a difference in their language development compared to those who do not^16^.

A suitable theory for the study is the Vygotsky Zone of Proximal Development (ZPD). This theory posits that parents and smart devices can scaffold the development of a child when placed in the right developmental level and ability of the child^17^. This scaffolding supports language development by making sure that media is right for the child’s environment of fast development can help children to learn better when parents actively take part in how their child uses smart devices^18^. Thus, Vygotsky’s ZPD offers a strong base to see if teaching how to use smart devices can affect young children’s language skills and if it can do so well or badly.

Vygotsky’s sociocultural concept emphasizes the relevant function of linguistic improvement and social interaction, and parental mediation in children’s cognitive development. Within the field of clever device use, certain empirical findings suggest that the satisfaction of parental involvement, joint attention, inclusive of scaffolding, and guided communication, considerably shapes how kids can practice and acquire language capabilities. Aslan and Turgut’s study, show that once parents actively mediate digital engagement, youngsters are more likely to benefit from the linguistic input embedded in apps and media, aligning with Vygotsky’s perception of the environment of better Development^12^. Conversely, passive tool use regularly correlates with weaker language results, underscoring the importance of parental scaffolding as a bridge between technological publicity and meaningful language acquisition.

### Empirical studies

The following studies aim to support this study so as to compare their results with this research results. Lame thinks that empirical studies are critical in a study since they provide a knowledge base, which helps clarify and define the research problem while preventing the researcher from duplicating efforts that were already done by other people and common mistakes made by other researchers^19^. Indeed, these works guide the improvement of the research methodology for the new study and its significance and credibility by placing it in the existing broader scholarship. It allows the researcher to make discernments about knowledge lacunae and build upon established findings, making valuable new contributions possible.

Rayce et al. studied whether there were relationships between mobile device screen time and lagging language development in toddlers. While very few studies exist concerning the use of mobile devices by young children, this could potentially impact language development. Rayce et al. conducted the present study in order to investigate potential relationships between mobile device screen time and both receptive language skills and expressive language skills. Data from the TRACES Danish nationwide survey (*n* = 31,125) for 2-and 3-year-old children was used in this study. Time spent daily using mobile devices was considered an indicator of mobile device screen time. It was through a multivariable linear regression analysis that we were able to see the relationship between the time children spent using their mobile devices and the development of language. Logistic regression analysis was used to see the risk of having significant problems with language. Findings indicated that an increased screen time of mobile devices of one hour was combined with low degrees of development in language, and the greater possibilities found for difficulties in understanding language and expressing language. Reading to the child does not affect any of these, except for the second one: it eases the difficulty in comprehending language^20^.

Çalhan and Göksu aimed to identify whether the parents’ role in media mediation with childhood children is associated with tendencies of digital game addictions. It was checked whether the variables were related to the habits of using digital tools by the child and the parent, and whether they changed according to different sociodemographic characteristics. Data were, therefore, obtained from 433 parents (mothers = 336, fathers = 97) of 3-6-year-old children. Screen time on a parent’s digital devices is negatively associated with parents’ role as mediators for digital media use and positively associated with children’s likelihood of being addicted to video games. Moms reported that their children exhibited more video game addiction than did dads. Kids whose parents played digital games had a greater propensity for digital game addiction than those whose parents did not; this was true for boys more so than girls. The children who owned personal tech tools had a greater propensity for digital game addiction than those who did not. Additionally, kids who watched YouTube clips had a greater propensity for digital game addiction than kids who only watched children’s TV programs. Finally, the data indicated that moms were more involved in the role of mediator for digital media use than were dads; however, parents acted more strictly toward their daughters^21^.

The Najiha et al.’s study analyzed how parents can help children’s language development in their infancy via mobile technology and Smartphone use. Communication is primarily achieved through language; thus, developing in children who have not yet learned the sounds of language, vocabulary of letters and words, and write together simple sentences is an extremely critical developmental milestone. The development of children’s language is the responsibility of parents, teachers, and digital media is a way to do so, specifically through media usage. The focus of the study is to explain the caregiver’s role in helping children’s language development through Smartphone media. The study uses a descriptive qualitative approach for its design. It was based on primary data collected from knowledgeable individuals or respondents (researchers); those knowledgeable individuals provided answers to a questionnaire that contained 2 yes/no questions, and several open-ended questions, which were obtained electronically via the Google Forms format. An analytical process, using an interactive model of analysis, was utilized. The three phases of the model were data reduction, validation, and data display. The study found that “wise” parental monitoring of children’s Smartphone use will stimulate children’s language development. The results of the study indicate that the caregiver plays a significant role in developing the children’s ability to communicate effectively and to think abstractly^15^.

Parents’ involvement in and their mediation of 0–3-year-olds’ digital play with smart devices is discussed in Nevski and Siibak’s study. This study discusses Estonian parents’ beliefs and actions regarding this issue. Parents whose 0–3-year-olds have been permitted to use smart devices (*n* = 198) were surveyed to examine if a relationship exists between the use of smart technology by the parents, the attitudes of parents toward the use of smart technology, and the age of the child that predicts the amount of smart device usage by young children as well as which types of smart devices are made available to 0–3-year-olds, how frequently the children may access these devices, and the types of activities and applications that the children engage in when using smart devices. Additionally, the authors assessed the various parental mediation methods used in relation to children’s digital play. The results indicate that parent screen time, parent attitudes, and the child’s age all influence the frequency of touch-screen device usage by 0–3 year olds, and also the reasons why parents allow children to use smart devices for educational or behavior regulation. Although parental mediation strategies vary based on the child’s age and sex; nevertheless, many parents report using a variety of strategies to provide adequate mediation for their child’s digital play^22^.

Moon conducted a cross-sectional study examining how young children’s use of smartphones/tablet computers relates to their language and development skills. The primary objective of the study was to identify any statistical associations between young children’s smartphone/tablet computer use and their overall cognitive/language abilities. Data for the study were obtained from parent questionnaire responses (regarding their child’s use of smart devices), and a child developmental screening instrument (a measure of children’s social development/social relationships; a measure of children’s language development/receptive-expressive language skills; and a measure of children’s fine motor skills). The sample consisted of 117 children who were enrolled in kindergarten in South Korea during the period of November 2015 through April 2016. There were three age groups included in the sample: children aged 3 years, children aged 4 years, and children aged 5 years. Based upon the results of the study, it was found that there was a statistically significant positive association between the amount of time spent using a smart device and the frequency of fine motor skill use among children aged 3 years (Spearman’s correlation coefficient = 0.426, *p* < 0.05). Similarly, the suitability of the children’s social relationships had a statistically significant positive association with the children’s social development at age 3 years (*p* < 0.05). Overall, the findings of the study suggest that smart device use is positively associated with children’s fine motor development at age 3 years, but negatively associated with children’s language development at age 3 years^16^.

## Methodology

### Research design

The study intends to look into how parents control the use of smart devices by young children and what it implies for their language skills. A quantitative approach is used. A quantitative approach as a methodology of research that concentrates on the gathering and examination of numerical data regarding patterns and hypothesis testing of relationships between variables. It employs structured tools like surveys, experiments, and statistical techniques to ensure objectivity and precision; findings can be applied to larger population groups^23^.

The study has an independent variable, and it is the parental mediation of smart device use. It is how parents manage, guide, or are otherwise involved with their child’s use of smart devices. It may consist of setting rules for the use of devices, co-using devices with the child (joint media engagement), setting restrictions on the type of content, and discussing the content with the child. The dependent variable is the development of language in early childhood, including vocabulary, grammatical rules, and social uses of language. Measurements of language development are usually conducted through standardized tests or checklists to determine the degree to which children can appropriately express ideas and comprehend spoken words at their age.

### Data collection methods and its procedures

After building and judging the questionnaire, it was distributed to the participants were chosen under the method of Stratified Sampling. The questionnaire was sent to the participants via social media such as WhatsApp and email. After filling the questions by the participants, their responses were analyzed by applying the SPSS program. According to Tong (2006), the Stratified Sampling Method is a probability sampling approach used in quantitative research. It requires the division of the population into distinct groups or strata in which predefined common characteristics are shared by the individuals. A random sample is then drawn from each of the groups by the researchers. This guarantees the presence of particular subgroups in the sample.

### Sampling method

The participants of this study were chosen using the Stratified Sampling Method. It is a probability sampling method used in quantitative research. The method used requires the population to be divided into distinct groups or strata where common pre-defined characteristics are shared by the individuals, and an element of each group is chosen randomly by the researchers^24^. This ensures the presence of some specific subgroups in the sample.

### Population and sample

The population of this study includes many families that have young children whose age is between 1 and 5 years old in Amman area. The sample consists of 82 families who are currently living in Amman area. The study sample size is adequate to ensure statistical reliability and validity.

### Research tool

The researcher developed a questionnaire of eight items that addressed the research questions for this study. The questionnaire has validity as it was evaluated by a group of university assistants and associate professors with similar majors (See Appendix A). In addition to validating the content, the judges made additions, deletions, or modifications to some items. The researcher developed the questionnaire to determine the ways that parents may be able to assist their young children to utilize smart devices and the effect on their language skills in Amman. In addition to content material validation via expert opinion, the researcher carried out a reliability analysis to assess the instrument’s internal consistency. He completed this evaluation by means of calculating Cronbach’s alpha coefficients for the entire device and its subscales. The consequences showed that the instrument’s reliability reached perfect to excellent levels, with Cronbach’s alpha coefficients above 0.8, indicating that the instrument gadgets continually measured the intended construct. This analysis ensured the device’s reliability beyond qualitative validation and highlighted the robustness of our records collection device. The exploratory issue evaluation was performed to determine the underlying element shape of the size. Subsequently, we carried out a confirmatory component analysis to verify that the proposed model provided a terrific match to the records. These two steps tested the assembly validity and dimensionality of our device for measuring national identification via early childhood education. The participants (82 families) filled out the questionnaire. Their answers are treated statistically. Likert’s five-point scale categorizes their responses as: ‘strongly disagree^1^’, ‘disagree^2^’, ‘neutral^3^’, ‘agree^4^, and ‘strongly agree^5^’. A Likert scale is defined as a rating that is used to measure opinions, attitudes, or behaviors^25^. Typically, a statement is given to which the individual has to express their degree of agreement or disagreement, with three or five alternatives.

### Data analysis

After developing and validating the questionnaire, it was filled out on a social media platform by the participants using WhatsApp and email. Data was collected and analyzed with SPSS, and results were attained from various statistical methods, such as a frequency descriptive test and a one-sample t-test. An analysis of the results was performed to attain the final results of this study. A few recommendations were made that could be used to conduct further studies in the future.

### Research results

The research question that guided the study is: How do parents mediate smart device use about its outcomes on language improvement in childhood in the Amman area? Three statistical tests were used to investigate the main research question. The tests is are: ‘Frequency Test’, ‘Descriptive Test’. And the ‘One-Sample T-Test’. The first test checks the responses’ percentage of the participants to the 8 items of this questionnaire (See Appendix A). The second test is the descriptive test, and the third statistical test (One-Sample T-Test) relates to the final result of the main research question.

**Table 1:** Participants’ valid percentage to the 8 items.

Degree of participants’ responses	The frequency of participants’ responses
Strongly disagree (1)	0.00%
Disagree (2)	7.2%
Neutral (3)	9.5%
Agree (4)	22.0%
Strongly agree (5)	61.3%
**Total**	**100.0%**

According to the participants’ responses, Table 1 clarifies that the average valid percent of the participants’ responses who answered with ‘strongly disagree’ is 0.00%. The average valid percent of the participants’ responses who answered with ‘disagree’ is 7.2%. The average valid percent of the participants’ responses who answered with ‘neutral’ is 9.5%. The average valid percent of the participants’ responses who answered with ‘agree’ is 22.0%. The average valid percent of the participants’ responses who answered with ‘strongly agree’ is 61.3%. Consequently, the average valid percent of the participants’ responses who answered with ‘strongly agree’ and ‘agree’ is 83.3%.

**Table 2:** Shows the responses of the participants regarding the 8 items.

	No. of participants	Minimum	Maximum	Mean		Std. Deviation
		Statistic	Statistic	Statistic	Std. Error	Statistic
**Item 1**	82	2.00	5.00	4.5000	0.07999	0.72436
**Item 2**	82	3.00	5.00	4.4878	0.07412	0.67117
**Item 3**	82	3.00	5.00	4.5610	0.07175	0.64974
**Item 4**	82	3.00	5.00	4.6220	0.07288	0.65997
**Item 5**	82	3.00	5.00	4.5244	0.07202	0.65217
**Item6**	82	3.00	5.00	4.5366	0.07402	0.67027
**Item 7**	82	3.00	5.00	4.5488	0.07187	0.65078
**Item 8**	82	3.00	5.00	4.5122	0.07612	0.68932
**Average**	82			**4.5366**		**0.67052**

The above Table 2 explains how parents mediate smart device use about its outcomes on language development in early childhood in the Amman area. These responses are to be analyzed using the Frequency and Descriptive Tests. Tables 1 and 2 show that the average percentage of the 8 items is nearly 83.3%. Besides, the average Mean of these items is 4.5366 with an average Standard Deviation of 0.67052. The Mean and the percentage of these 8 items are nearly high. According to the participants’ responses, guiding a child’s use of smart devices by setting clear rules and limits on screen time is significantly useful. The process of monitoring the content a child accesses on smart devices positively supports learning and language development. Engaging with a child while using a smart device significantly encourages conversation and language-rich interactions. Parents do not believe that sitting with their children and interacting with them while using smart devices allows them to learn better linguistics and understand and use language in context. Parents agree that too much unsupervised use of smart devices can cause problems for children’s concentration and expressive language skills. Finally, there is agreement that using parental controls and passwords on smart devices helps manage the access of children to that content, hence providing a safer environment for language learning.

To elicit the final results of this main research question, One Sample T-test is conducted.

**Table 3:** Conducting a one sample T-Test to elicit the final results.

	No. of participants	Average Mean	Average Std. Devia- Tion	Sig. (2-tailed)	Average percentage
Items: 1–8	82	4.5366	0.67052	0.000	83.3%
Total	82				

Table 3 shows this. Since the average Mean is nearly high (4.5366), the percentage is 83.3%, and the Significance (2-tailed) is 0.000, it means that there is no active control and guidance by parents in Amman over the use of smart devices by young children because of the structured rules that monitor and, as a result, control the screen time, which may have negative impacts on the development of children’s language. The results of the study indicate the high importance of the need for educational programs for Amman parents and parents everywhere. This ensures that there are effective strategies for active mediation beyond mere rules of screen time.

## Discussion

The results of this study in Amman found a worrying shortage of active parental control and guidance over the use of smart devices by little children. It seems that there is an over-reliance on structured rules to monitor screen time rather than the actual interaction between the parent and the child. At best, this best passive form of supervision could be particularly problematic considering the potential negative effects of screen time on language development. The results highlight a discrepancy between the perceived notion of control through setting rules and the actual realization, which entails active engagement. The process of limiting screen time won’t be sufficient to mitigate the adverse effects on the growth of children’s linguistic growth.

The study results also showed that there was general disagreement between the surveyed parents on whether the use of content on the smart device was going to add value to the early reading and language skills of the child, even if the use is appropriate and under supervision. These align with Najiha et al’s^15^ findings, which strongly established the downside of smart devices in promoting linguistic development through smartphone media, specifically indicating benefits under supervision on wide thinkers and skillful communicators. This would mean, thus, that the better part of the parents in Amman are not exploiting this active engagement to the maximum. This study also concurs with that of Nevski and Siibak (2020), where intensive statistical relations were found between parent screen time, parental attitudes, child’s age, and touch screen use, influenced by parental motives associated with education, entertainment, and control over behavior. This coming together of results shows a bigger difficulty in how parents act toward screen content, where there is a greater and more aware role needed to help good growth results for little kids.

### The implications of the findings

The findings of this study carry high significance of the need for educational programs not only for Amman parents but parents everywhere, underlining effective strategies for active mediation beyond mere rules of screen time. Results implied that current attitudes of parents toward content and co-engagement of smart devices may hinder maximum language acquisition by the child; there was a disconnect between perceived benefits and actual developmental outcomes. Therefore, policymakers and educators need to have programs that can empower parents with knowledge and skills to turn passive screen time into active learning episodes that could veritably support early language development.

### Limitations of the study

An important limitation of the study is that it relies on the data provided by the parents; therefore, responses may be biased due to factors such as social desirability. It shall also hinder an objective assessment of the actual language development on the part of the children. Moreover, the results are confined to the perceptions of parents from Amman, and it may not be applied to other regions or different socio-cultural contexts within Jordan or in the world at large.

### Gap identification

The specific effective strategies of parental mediation for smart device use that actively foster language development in early childhood, particularly within the Jordanian cultural context, remain a significant gap, which the growing body of research on smart devices has not successfully filled. Most existing literature often does not paint a detailed action how the parents can effectively mediate to maximize developmental benefits and mitigate risks of l9anguageacquisitionquisition by the child and therefore retain less value, nurturance more than screen time, on the impact of smart devices. language development.

## Conclusion

This study has indicated that the parental mediation of smart device use throughout early childhood in Amman is more characterized by passive control, such as rules of screen time, rather than active participation and instruction. Lack of agreement between parents on the didactic value of the digital content in the development of linguistic skills and relatively low belief in the effectiveness of co-interaction during the time the child spends looking at the screen seems to impede the best linguistic outcome. These results are consistent with similar international research, though on much broader parameters. At the most, the study has brought out a critical gap in the existing parental mediation practice in this particular context to smart devices becoming truly supportive, rather than potentially inhibitive, of early language development.

### Recommendations

Based on the results, the subsequent efforts should focus on making a change by developing and implementing specific educational programs for parents in Jordan. The programs should highlight the critical role of active parental mediation, including co-viewing and interactive engagement while using the smart device, to effectively stimulate early language development. Partnerships between policymakers, educational institutions, and other relevant stakeholders to develop guidelines and resources for parents to critically assess digital content and turn the screen time into a meaningful learning process will promote healthier digital habits and linguistic growth in children.

Here is a methodological enhancement to address limitations:

The researcher can use a longitudinal, mixed‑methods design with diverse sampling (urban/rural, SES), objective usage data (time‑use, device analytics diaries), multi‑informant reports, standardized language (teachers/parents), and transparent practices (open data/materials, pre-registration) to strengthen generalizability and validity.

## Supplementary Information

Below is the link to the electronic supplementary material.

Supplementary Material 1

## Data Availability

The datasets generated and analyzed during the current study are not publicly available due to confidentiality agreements with the participating families. Anonymized data may be made available from the corresponding author upon reasonable request and subject to institutional approval.
