# Supplementary material for: Parental mediation of smart device use and its impact on language development in early childhood
Source: Sci Rep. 2026 Feb 10;16:8209. doi: 10.1038/s41598-026-38833-9 (PMC12963561; doi:10.1038/s41598-026-38833-9)
Supplement: Supplementary file 1 — Supplementary Material 1 [file 41598_2026_38833_MOESM1_ESM.docx]

**Appendix A**

**Dear families in Amman area**

This study investigates how parents guide young children's use of smart devices and what it means for their language skills in Amman area.

Please read the following items and share your opinion by checking the appropriate box. Your responses are greatly appreciated for conducting this study.

| **No.** | **Items** | **Strongly**  **agree** | **Agree** | **Neutral** | **Disagree** | **Strongly**  **dissagree** |
| --- | --- | --- | --- | --- | --- | --- |
| **1.** | Guiding a child’s use of smart devices should be done by setting clear rules and limits on screen time. |  |  |  |  |  |
| **2.** | Monitoring the content a child accesses on smart devices supports learning and language development. |  |  |  |  |  |
| **3.** | Parents do not believe that sitting with their children and interacting with them while using smart devices allows them to learn better linguistics and understand and use language in context. |  |  |  |  |  |
| **4.** | Parents agree that too much unsupervised use of smart devices can cause problems for children’s concentration and expressive language skills. |  |  |  |  |  |
| **5.** | Parents know how to use smart devices as supplements to traditional talk, such that a balance between screen and non-screen time is maintained, thereby producing healthy linguistic and social results. |  |  |  |  |  |
| **6.** | Engaging with a child while using a smart device can encourage conversation and language-rich interactions. |  |  |  |  |  |
| **7.** | Parents agree that cutting down screen time allows more face-to-face interactions, which are the best way to develop pragmatic and social language. |  |  |  |  |  |
| **8.** | There is agreement that using parental controls and passwords on smart devices helps manage the access of children to that content, hence providing a safer environment for language learning. |  |  |  |  |  |
